# Immobilized *Rhizopus oryzae *lipase catalyzed synthesis of palm stearin and cetyl alcohol wax esters: Optimization by Response Surface Methodology

**DOI:** 10.1186/1472-6750-11-68

**Published:** 2011-06-17

**Authors:** Mohamed Sellami, Imen Aissa, Fakher Frikha, Youssef Gargouri, Nabil Miled

**Affiliations:** 1 Laboratoire de Biochimie et de Génie Enzymatique des Lipases, ENIS, Université de Sfax, route de Soukra, BPW 1173 Sfax-Tunisia

## Abstract

**Background:**

Waxes are esters of long-chain fatty acids and long-chain alcohols. Their principal natural sources are animals (sperm whale oil) and vegetables (jojoba) which are expensive and not easily available. Wax esters synthesized by enzymatic transesterification, using palm stearin as raw material, can be considered as an alternative to natural ones.

**Results:**

Palm stearin is a solid fraction obtained by fractionation of palm oil. Palm stearin was esterified with cetyl alcohol to produce a mixture of wax esters. A non-commercial immobilized lipase from *Rhizopus oryzae *was used as biocatalyst. Response surface methodology was employed to determine the effects of the temperature (30-50°C), the enzyme concentration (33.34-300 IU/mL), the alcohol/palm stearin molar ratio (3-7 mol/mol) and the substrate concentration (0.06-0.34 g/mL) on the conversion yield of palm stearin. Under optimal conditions (temperature, 30°C; enzyme concentration, 300 IU/mL; molar ratio 3 and substrate concentration 0.21 g/mL) a high conversion yield of 98.52% was reached within a reaction time of 2 h.

**Conclusions:**

Response surface methodology was successfully applied to determine the optimum operational conditions for synthesis of palm stearin based wax esters. This study may provide useful tools to develop economical and efficient processes for the synthesis of wax esters.

## Background

Palm oil is one of the traditional fats that have been widely used throughout the world in human diet. Global palm oil production was estimated to 42 million ton during 2007/2008, accounting for 40% of the worldwide production of total dietary oils [1]. Palm oil contains a mixture of high and low melting triglycerides. By a simple dry fractionation process under a controlled temperature, palm oil can be resolved into a liquid (olein) and a solid (stearin) fraction [2]. The palm olein fraction is the mostly used in industry due to its low melting point [3]. The high melting point of palm stearin (44-56°C) poses problems in manufacturing of edible fats such as margarine and shortenings as it confers low plasticity to the end product. Many attempts have been carried out to maximise the use of palm stearin by transesterification [4-7]. Among transesterification reactions, alcoholysis of triacylglycerols to produce wax esters is relatively a simple process and rather economically important to the oil and fat industries [8].

Waxes are esters of long-chain fatty acids and long-chain alcohols. The principal natural sources of waxes are from animals and vegetables [9]. They can be composed of saturated and unsaturated wax esters. The saturated wax esters are predominant in beeswax, whereas, traditional raw materials for unsaturated wax esters are sperm whale and jojoba oil. Commercial waxes have a wide range of applications as lubricants, polishes, plasticizers, coating materials in the pharmaceutical and food industries. They are also used as ingredients in the formulation of cosmetics and other chemical products [10]

Enzymatic synthesis of wax esters from different raw materials have been reported [8,10-12]. In these studies, high yields of palm oil based wax esters have been obtained. Few studies reported the use of palm stearin and oleyl alcohol to generate wax esters [13]. The stearin fraction of palm oil is technologically underutilized because of its high melting point. The objective of our study was to promote the use of the palm stearin fraction of palm oil in wax esters synthesis. Furthermore, this is to our knowledge the first study of using cetyl alcohol and palm stearin to synthesize wax esters by means of response surface methodology.

Enzymatic synthesis of wax esters from palm stearin and cetyl alcohol, a typical fatty alcohol present in natural waxes [11], was carried out using a non commercial immobilised lipase from *Rhizopus oryzae *[14,15]. A three-level four-factor central composite rotatable design was adopted in our study to determine the optimum conditions. This method is a useful technique to predict the best performance conditions with a reduced number of experiments [16,17].

## Methods

### Fractionation process

Refined, bleached and deodorized palm oil, of iodine value 53.0, was obtained from the Tunisian Olive Oil Office. It was fractionated in the laboratory using a dry fractionation process, according to the method described by Thiagarajah [18]. Palm oil was melted and kept homogenized at 70°C to destroy all crystals present. The melted oil was agitated with stirring rate of 25 rpm. A fractionation temperature of 30°C was used. After stabilization, two fractions were obtained, namely stearin (solid) and olein (liquid). They were separated by vacuum filtration. The palm stearin fraction was used to produce wax esters.

### Production and immobilisation of lipase

*Rhizopus oryzae *lipase was produced as described by Ben salah et al [14]. The enzyme immobilization was made onto CaCO_3 _as described by Ghamgui et al [15]. The activity of immobilized lipase was measured titrimetrically with a pH-stat, under the standard assay conditions described previously by Rathelot et al [19] using olive oil (10%) emulsion as substrate. One international unit (IU) of lipase activity was defined as the amount of lipase that catalyzes the liberation of 1 μmol of fatty acid per min at pH 8.5 and 37°C.

### Experimental design

Response Surface Methodology was applied to establish the relationship between the response studied (conversion yield of palm stearin) and the four selected experimental variables (temperature; enzyme concentration; substrate molar ratio (cetyl alcohol/palm stearin) and substrate concentration). A quadratic polynomial model has been postulated and represented by the following equation:

Where:  is the conversion yield; b_0_, b_j_, b_jk _and, b_jj _are the estimated model coefficients and X_j _is the coded variables. In this study, the generation and the data treatment of the composite design are performed using Design-Expert 7 software (Stat-Ease Inc., USA).

### Transesterification reactions

According to the experimental design (Table 1), the transesterification reactions were carried out in screw-capped flasks containing different amount of cetyl alcohol (from Sigma) and palm stearin (MW = 3 × average of saponification equivalent of palm stearin = 600 g/mol). 3 mL of *n*-hexane were added in all reaction mixtures. Different concentrations of immobilized lipase were also added in the mixtures with respect to the reaction conditions in Table 1. The reaction mixture was incubated at different temperatures and shaked (200 rpm). A control reaction without adding enzyme was carried out in parallel. After a reaction time of 2 h, 200 μl aliquots of the reaction mixture were withdrawn and the immobilized enzyme was removed by centrifugation at 15,000 rpm (2,400 g) for 5 min. The supernatant was used to analyze wax esters yields. All samples were assayed in duplicates to reduce inter-assay variations and the mean values of the replicates were reported.

**Table 1:** Experimental conditions of the composite design and the corresponding experimental response

Experimental conditions					Response	
**Run**	**Temperature (°C)**	**Enzyme Concentration (IU/mL)**	**Molar ratio (mol/mol)**	**Substrate Concentration (g/mL)**	**Actual yield (%)**	**Predicted yield (%)**

**1**	50 (+1)	300 (+1)	7 (+1)	0.06 (-1)	99.51	96.52
**2**	50 (+1)	300 (+1)	3 (-1)	0.06 (-1)	73.15	75.97
**3**	50 (+1)	33.34 (-1)	7 (+1)	0.34 (+1)	44.41	41.42
**4**	30 (-1)	300 (+1)	3 (-1)	0.34 (+1)	52.86	55.68
**5**	50 (+1)	33.34 (-1)	3 (-1)	0.34 (+1)	11.03	13.85
**6**	30 (-1)	33.34 (-1)	7 (+1)	0.06 (-1)	19.54	16.55
**7**	30 (-1)	300 (+1)	7 (+1)	0.34 (+1)	54.45	51.46
**8**	30 (-1)	33.34 (-1)	3 (-1)	0.06 (-1)	32.84	35.66
**9**	30 (-1)	166.67 (0)	5 (0)	0.2 (0)	84.92	85.28
**10**	50 (+1)	166.67 (0)	5 (0)	0.2 (0)	86.87	87.23
**11**	40 (0)	33.34 (-1)	5 (0)	0.2 (0)	20.065	20.21
**12**	40 (0)	300 (+1)	5 (0)	0.2 (0)	88.61	88.97
**13**	40 (0)	166.67 (0)	3 (-1)	0.2 (0)	53.74	42.47
**14**	40 (0)	166.67 (0)	7 (+1)	0.2 (0)	36.69	48.67
**15**	40 (0)	166.67 (0)	5 (0)	0.06 (-1)	76.41	76.77
**16**	40 (0)	166.67 (0)	5 (0)	0.34 (+1)	42.25	42.61
**17**	40 (0)	166.67 (0)	5 (0)	0.2 (0)	62.45	65.97
**18**	40 (0)	166.67 (0)	5 (0)	0.2 (0)	76.22	65.97
**19**	40 (0)	166.67 (0)	5 (0)	0.2 (0)	66.51	65.97
**20**	40 (0)	166.67 (0)	5 (0)	0.2 (0)	64.78	65.97
**21**	40 (0)	166.67 (0)	5 (0)	0.2 (0)	62.02	65.97

### TLC Analysis

The reactants were separated by thin-layer chromatography (TLC) at room temperature according to Keng et al [20]. After elution, the plates were dried and stored in an iodine chamber. The presence of the cetyl alcohol, palm stearin fractions and their corresponding esters were detected as brown spots and were identified as compared to authentic standards. Wax standards used were: cetyl oleate, cetyl palmitate, cetyl linoleate and cetyl stearate synthesized in our laboratory. Quantification of the TLC plate was accomplished with the software MCID Analysis v7.0 from Imaging Research (St. Catherine, Ontario, Canada). The ratio: Optical density values/spot area for palm stearin was measured by the MCID software. The initial (t = 0 h) optical density/spot area value corresponds to 100% of the substrate. The residual optical density/spot area value (expressed as percentage of the initial value) measured at the end of the reaction corresponds to the residual substrate (expressed as percentage of the initial substrate concentration). The conversion yield corresponds to (100 - residual optical density/spot area value).

### HPLC Analysis

The identification of wax esters was carried out by HPLC analysis. It was performed on a Perkin Elmer apparatus composed of Series 200 UV/Vis detector and Series 200 Micro Pump. The column was a Lichrospher 100 RP-18, 5 μm (4 × 250 mm), and its temperature was maintained at 45°C. The flow rate was 1.5 mL/min. The mobile phase used was acetonitrile/acetone (50:50, v/v) for a total running time of 35 min. Peak detection was monitored at 206 nm [12].

## Results and discussion

Immobilized *Rhizopus oryzae *lipase was used to catalyze the synthesis of wax esters. The addition of hexane to the reaction medium was found necessary to improve the stability of the immobilized *Rhizopus oryzae *lipase and the solubility of the substrates (data not shown). Furthermore, the addition of water at the beginning of the reaction failed to improve the conversion yield. In the light of preliminary studies (data not shown), the temperature, the enzyme concentration, the alcohol/TG molar ratio and the substrate concentration which were the most effective on the response, were chosen as operating variables (X_1_, X_2_, X_3 _and X_4_, respectively) to optimize the synthesis yield.

### Model fitting and ANOVA

To carry out a cuboidal composite experimental design, a high and a low level were chosen for each factor (Table 2). Table 1 shows the real experimental conditions of the composite design with the corresponding measured responses. The results were analyzed using a multi regression method [21].

**Table 2:** Variables values used in the Experimental Design

Factor	Variables	Unit	level		
			-1	0	1
**Temperature**	X_1_	°C	30	40	50
**Enzyme Concentration**	X_2_	IU/mL	33.34	166.67	300
**Molar ratio (Cetyl alcohol/Palm stearin)**	X_3_	mol/mol	3	5	7
**Substrate Concentration**	X_4_	g/mL	0.06	0.2	0.34

Fitting the data to various models (linear, two factorial, quadratic and cubic) and their subsequent ANOVA showed that reactions of palm stearin and cetyl alcohol were most suitably described using a quadratic polynomial model (Table 3). The second-order polynomial equation generated by the Design-Expert software is:  = 65.97 + 0.97 X_1 _+ 34.18 X_2 _+ 3.10 X_3 _- 17.08 X_4 _- 9.29 X_1_X_2 _+ 8.93 X_1_X_3 _+ 12.66 X_1_X_4 _+ 0.98 X_2_X_3 _- 7.58 X_2_X_4 _+ 2.74 X_3_X_4 _+ 20.28 X_1_^2^-11.18 X_2_^2 ^- 20.40 X_3_^2 ^-6.28 X_4_^2^, where X_1_, X_2_, X_3 _and X_4 _are the coded variables.

**Table 3:** Model Summary Statistics

Source	Standard deviation	R-Squared	Adjusted R-Squared	Predicted R-Squared	PRESS
**Linear**^***a***^	17.050	0.611	0.514	0.232	9179.793
**2FI**	18.598	0.711	0.421	-5.630	79274.413
**Quadratic**^***a***^	8.884	0.960	0.868	-5.867	82113.127
**Cubic**^***b***^	5.785	0.989	0.944		+

2FI = two factor interaction;

PRESS = predicted residual error sum of squares;

*a*: suggested model to be selected;

*b*: aliased model not to be selected

+ Case with leverage of 1: PRESS statistic not defined.

In order to determine the adequacy of the quadratic model, an analysis of variance (ANOVA) was conducted using the usual Fisher's *F*-tests (Table 4), in which the regression sum of squares is subdivided into two parts. One attributed to the linear regression and the other to the quadratic model [22].

**Table 4:** ANOVA and Regression Analysis of the Quadratic Model

Source of variation	Sum of Squares	Degrees of freedom	Mean Square	*F*-value	*p*-value Prob > F	Signif
**Model**	**11483.454**	**14**	**820.247**	**10.392**	**0.0044**	***a***
X_1_	1.901	1	1.901	0.024	0.8818	
X_2_	2336.545	1	2336.545	29.603	0.0016	
X_3_	95.976	1	95.976	1.216	0.3124	
X_4_	583.453	1	583.453	7.392	0.0347	
X_1 _X_2_	138.198	1	138.198	1.751	0.2340	
X_1 _X_3_	638.138	1	638.138	8.085	0.0294	
X_1 _X_4_	256.492	1	256.492	3.250	0.1215	
X_2 _X_3_	7.742	1	7.742	0.098	0.7647	
X_2 _X_4_	91.839	1	91.839	1.164	0.3222	
X_3 _X_4_	60.006	1	60.006	0.760	0.4168	
X_1_^2^	1050.075	1	1050.075	13.304	0.0107	
X_2_^2^	319.292	1	319.292	4.045	0.0910	
X_3_^2^	1062.246	1	1062.246	13.458	0.0105	
X_4_^2^	100.796	1	100.796	1.277	0.3016	
**Residuals**	**473.577**	**6**	**78.929**			
Lack of Fit	339.721	2	169.861	5.076	0.0799	***b***
Pure Error	133.856	4	33.464			
**Cor Total**	**11957.031**	**20**				
**R-Squared = 0.960**						

*a*: indicates significant at the level of 99%

*b*: indicates insignificant at the level of 99%

Estimated standard deviation is equal to Std. Dev. = √473.577/6 = 8.884

The regression sum of squares was statistically significant when using the *F*-test at a 99% probability level. This suggests that the variation taken into account by the model was significantly greater than the unexplained variation. Likewise, the statistically insignificant lack of fit and the satisfactory levels of R-Squared (0.96) indicated that the model was statistically significant and adequate to represent the relationship between the experimental parameters.

Coefficients of the model were evaluated by regression analysis and tested for their significance (Table 4). *p*-values lesser than 0.0500 indicates that the model terms are significant. Among these parameters, the enzyme concentration (*p*-value = 0.0016) and substrate concentration (*p*-value = 0.0347) have highly significant effects (according to their small *p*-values) on the synthesis of wax esters as compared to the temperature and the alcohol/palm stearin molar ratio. Interactions between temperature and molar ratio alcohol/palm stearin (*p*-value = 0.0294) on one hand and substrate concentration and temperature on the other hand (*p*-value = 0.1215) were found to be modestly significant.

### Graphical Interpretation of the Response Surface Model

Relationships between the response and the experimental parameters were explored using contour plots. The response surface and the isoresponse curves were plotted against two variables while the two others were held constant at their mean levels.

**Figure 1:**
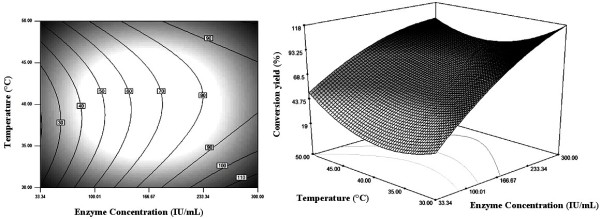
**Contour and response surface plots showing the effect of enzyme concentration and temperature on the synthesis yield of palm stearin based wax esters**. Substrate concentration and alcohol/palm stearin molar ratio were at their zero levels.

Figure 1 represents the effect of enzyme concentration and reaction temperature on the conversion yield of palm stearin.

Ester synthesis increased rapidly with increasing the enzyme amount. However, the reaction temperature did not have an appreciable influence on ester synthesis. A high conversion yield (>90%) was reached by using a high enzyme concentration (>233 IU/mL) for a very large range of temperatures.

**Figure 2:**
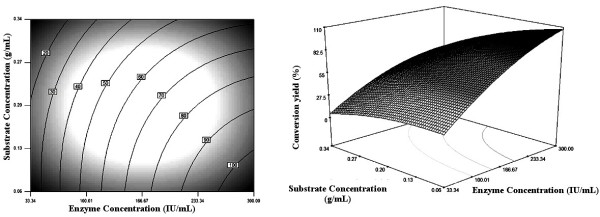
**Contour and response surface plots showing the effect of enzyme and substrate concentrations on the synthesis yield of palm stearin based wax esters**. Temperature and alcohol/palm stearin molar ratio were kept at their zero levels.

Both enzyme and substrate concentrations seem to play an important role in enhancing synthesis yield of palm stearin based wax esters (figure 2). A significant decrease in the conversion yield was noticed when using a high substrate concentration at any enzyme concentration. On the contrary, a high conversion yield was accomplished by using a low substrate concentration (around 0.06 g/mL) and a high enzyme concentration (>233 IU/mL). These findings are in line with previous reports that the presence of a high substrate concentration could have a negative effect on lipase catalyzed synthesis activity [23,24]. Besides, alcohols are known to be terminal inhibitors of lipases [10,25]. Furthermore, high amount of palm stearin would lead to a greater resistance to mass transfer because of a higher viscosity of the medium [26].

**Figure 3:**
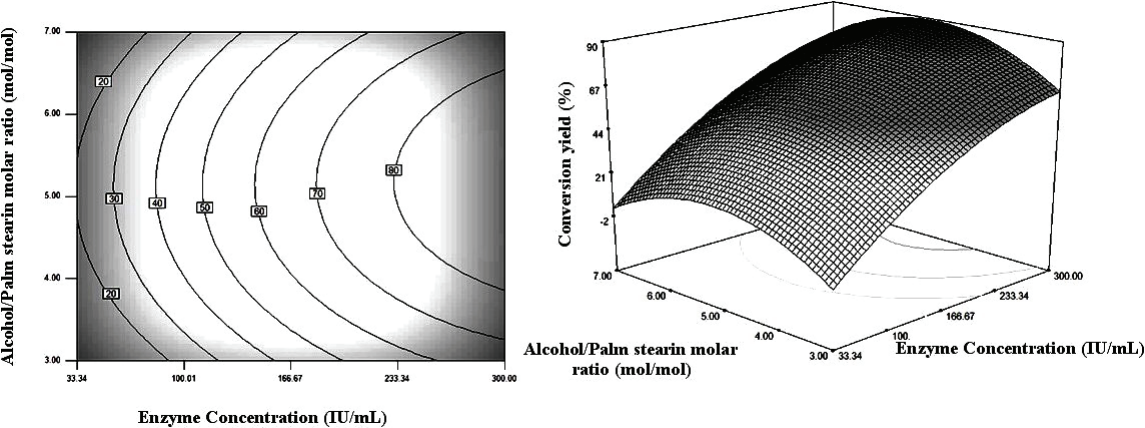
**Contour and response surface plots showing the effect of enzyme concentration and alcohol/palm stearin molar ratio on the synthesis of palm stearin based wax ester**. Temperature and substrate concentration were constant at their zero levels.

Figure 3 represents the effect of varying enzyme concentration and alcohol/palm stearin molar ratio on the conversion yield of palm stearin. The wax esters synthesis increased markedly with the enzyme concentration while alcohol/palm stearin molar ratio showed parabolic effects on the reaction yield (figure 3). Many lipases exhibit this type of dome shaped plots when catalyzing esterification reactions [27]. The optimum value of alcohol/palm stearin molar ratio was around 5 mol/mol (figure 3). A reaction with a moderate cetyl alcohol/palm stearin molar ratio and a high enzyme concentration (>233 IU/mL) achieved the maximal conversion yields (>80%).

**Figure 4:**
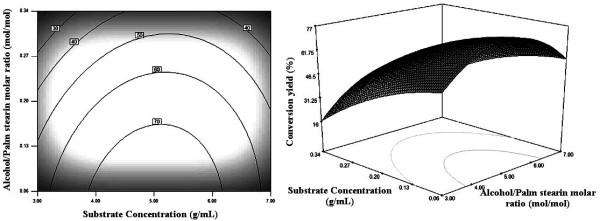
**Contour and response surface plots showing the effect of alcohol/palm stearin molar ratio and substrate concentration on the synthesis yield of palm stearin based wax ester**. The enzyme concentration and temperature were maintained at their zero levels.

Figure 4 represents the effect of varying substrate concentrations and alcohol/palm stearin molar ratios on the conversion yield of palm stearin. Increasing the alcohol/palm stearin molar ratio from 3 to 5 could enhance the conversion yield. Rising alcohol/palm stearin ratio value beyond 5 caused the reaction conversion yield to decline. This phenomenon also reported by Karboune et al [28] for the lipase-catalyzed transesterification of long-chain triglycerides with phenolic acid, might be attributable to a great resistance of mass transfer due to a high substrate concentration. A high conversion yields could be reached when minimizing the substrate concentration and using an alcohol/palm stearin molar ratio around 5 mol/mol.

### Optimal reaction conditions

The optimal synthesis conditions of palm stearin based wax esters were predicted using the optimization function of the Design Expert Software. These optimal conditions are presented in table 5 along with their experimental and predicted values. A good correlation was found between predicted and experimental values implying that empirical models derived from response surface methodology can adequately describe the relationship between the factors and their influence on the palm stearin based wax esters synthesis.

**Table 5:** Solutions for optimal conditions as generated by the Design Expert Software

Experiment	Temperature (°C)	Enzyme Concentration (IU/mL)	Molar ratio	Substrate Concentration (g/mL)	Experimental yield (%)	Predicted yield (%)	Standard deviation	**t**_**exp**._	Degrees of freedom	Signif
1	30	131.73	4.92	0.06	93.24	94.91	8.88	0.150	6	*a*
2	30	300	3.00	0.21	98.52	99.45	8.88	0.081	6	*a*
3	50	246.07	5.43	0.26	95.44	93.93	8.88	0.131	6	*a*

*a*: Indicate insignificant at the level of 99%

**Figure 5:**
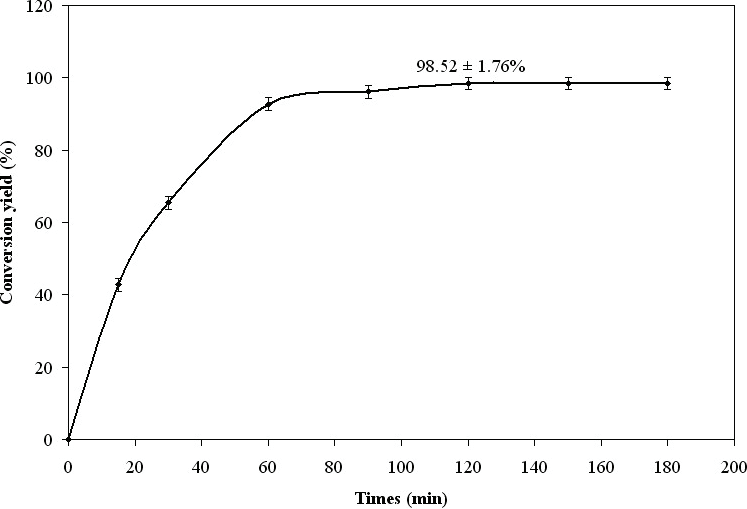
**Production of palm stearin based wax ester during alcoholysis reaction under optimal condition**. Temperature, 30°C; enzyme concentration, 300 IU/mL; molar ratio, 3 and substrate concentration, 0.21 g/mL.

Among the various optimum conditions, the highest conversion yield was obtained for experiment 2, which is carried out at 30°C, using 300 IU/mL of enzyme, an alcohol/triglyceride molar ratio of 3 and a substrate concentration of 0.21 g/mL. Under these optimal conditions, the time course of the alcoholysis reaction between the palm stearin and the cetyl alcohol was presented in figure 5. The conversion yield increased rapidly to reach its maximal value of 98.52 ± 1.76% within 2 h of reaction time. The experimental optimal synthesis yield value was close to the calculated one (99.45%). *Rhizopus oryzae *lipase immobilized on CaCO_3 _used in this work shows an interesting behavior by providing a high esterification yield (98.52%) within 2 h of reaction time. A comparable synthesis yield (92.3%) was obtained by Keng et al [29], but within a longer reaction time (5 h) when using palm oil and oleyl alcohol as substrates and Lipozyme as biocatalyst. Nevertheless, much lower synthesis yields were reported by Gunawan et al [8] (84.6% within 7.38 h) when esterifying oleyl alcohol and palm oil by Lipozyme, used as biocatalyst. It's worth to notice that theses studies were also optimized using RSM methodology.

### TLC and HPLC analysis of palm based wax esters under optimal conditions

**Figure 6:**
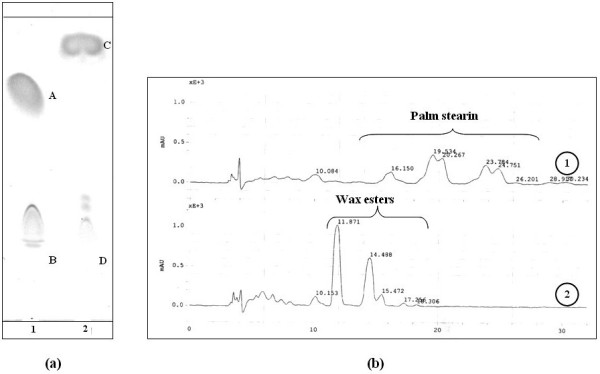
**(a) Thin layer chromatogram of palm stearin based, wax ester synthesis**. Mobile phase: hexane/diethyl-ether/acetic acid 90:10:0.5 (v/v/v). Lane 1: mixture before reaction showing palm stearin (A) and cetyl alcohol (B); lane 2: mixture after 2 h of reaction time showing wax esters (C) and residual cetyl alcohol (D). (b) HPLC profiles of palm stearin/cetyl alcohol mixture obtained before at 0 h (1) and 2 h (2) of reaction times. The column used was: Lichrospher 100 RP-18. 5 μm (4 × 250 mm) and the mobile phase used was acetonitrile/acetone (50:50. v/v) with a flow rate of 1.5 mL/min. Temperature was maintained at 45°C and UV detection was at 206 nm.

Figure 6a depicts the TLC analysis of palm stearin wax esters under optimal synthesis conditions. Upon a synthesis reaction time of 2 h, spots corresponding to alcohol and triglycerides disappeared, concomitantly with an appearance of a new band corresponding to wax esters.

HPLC analysis of palm stearin wax esters (figure 6b) was conducted and identification of peaks was carried out by comparing the chromatograms to those of authentic standard esters. The four main wax esters were cetyl palmitate, cetyl stearate, cetyl oleate and cetyl linoleate corresponding to retention times of 15.472, 18.306, 11.871 and 14.488 min, respectively. The peaks corresponding to triglycerides disappeared indicating they were all transesterified with cetyl alcohol to produce wax esters. These results are in agreement with the measured high conversion yields (Table 5).

## Conclusions

The enzymatic synthesis of palm stearin based wax esters by means of esterification with cetyl alcohol using an immobilized *Rhizopus oryzae *lipase as a biocatalyst was carried out in *n*-hexane. Response surface methodology was successfully applied to determine the optimum operational conditions for maximal conversion yields of palm stearin. The greatest conversion yields were obtained for a high enzyme concentration, a moderate alcohol/triglyceride molar ratio and a low substrate concentration. For example, a high conversion yield of 98.52% was reached within 2 h at 30°C, using an enzyme concentration of 300 IU/mL, an alcohol/triglyceride molar ratio of 3 and a substrate concentration of 0.21 g/mL. This study may provide useful tools to develop economical and efficient processes for the synthesis of wax esters.

## Competing interests

The authors declare that they have no competing interests.

## Authors' contributions

MS and IA designed the experiments, carried out the synthesis and the analysis of wax esters and drafted the manuscript. FF carried out the statistical analysis. YG and NM have conceived research and approaches and have given final approval of the version to be published. All authors read and approved the final manuscript.

## References

[B1] FAOSTAT Online Statistical Service United Nations Food and Agriculture Organization (FAO) 2009 http://faostat.fao.org 19655658

[B2] Zaliha O Chong CL Cheow CS Norizzah AR Kellens MJ Crystallization properties of palm oil by dry fractionation Food Chemistry 2004 86 2 245 250 10.1016/j.foodchem.2003.09.032

[B3] Lai OM Ghazali HM Chong CL Use of enzymatic transesterified palm stearin-sunflower oil blends in the preparation of table margarine formulation Food Chemistry 1999 64 1 83 88 10.1016/S0308-8146(98)00083-1

[B4] Lai OM Ghazali HM Cho F Chong CL Enzymatic transesterification of palm stearin: anhydrous milk fat mixtures using 1,3-specific and non-specific lipases Food Chemistry 2000 70 1 221 225

[B5] Graille J Pina M Moutet D Muderhwa JM Making value-added products from palm oil by 1.3-regioselectivity enzymatic interesterification ELAIES 1992 4 1 10

[B6] Lai OM Ghazali HM Chong CL Effect of enzymatic transesterification on the melting points of palm stearin:sunflower oil mixture Journal of the American Oil Chemists Society 1998 75 881 886 10.1007/s11746-998-0241-2

[B7] Lai OM Ghazali HM Chong CL Physical properties of *Pseudomonas *and *Rhizomucor miehei *lipase-catalyzed transesterified blends of palm stearin: palm kernel olein Journal of the Oil Chemists Society 1998 75 953 959

[B8] Gunawan ER Basri M Abd Rahman MB Salleh AB Abd Rahman RNZ Lipase catalyzed synthesis of palm-based wax esters Journal of oleo science 2004 53 471 477 10.5650/jos.53.471

[B9] Patel S Nelson DR Gibbs AG Chemical and physical analyses of wax ester properties Journal of Insect Science 2001 1 4 11 15455064 10.1673/031.001.0401 PMC355888

[B10] Gunawan ER Basri M Abd Rahman MB Salleh AB Abd Rahman RNZ Study on response surface methodology (RSM) of lipase-catalyzed synthesis of palm-based wax ester Enzyme and Microbial Technology 2005 37 7 739 744 10.1016/j.enzmictec.2005.04.010

[B11] Salis A Solinas V Monduzzi M Wax esters synthesis from heavy fraction of sheep milk fat and cetyl alcohol by immobilised lipases Journal of Molecular Catalysis B: Enzymatic 2003 21 4-6 167 174 10.1016/S1381-1177(02)00124-8

[B12] Decagny B Jan S Vuillemard JC Sarazin C Séguin JP Gosselin C Barbotin JN Ergan F Synthesis of wax ester through triolein alcoholysis: Choice of the lipase and study of the mechanism Enzyme and Microbial Technology 1998 22 7 578 582 10.1016/S0141-0229(97)00240-8

[B13] Keng PS Basri M Zakaria MRS Abd Rahman MB Ariff AB Abd Rahman RNZ Salleh AB Newly synthesized palm esters for cosmetics industry Industrial crops and products 2009 29 37 44 10.1016/j.indcrop.2008.04.002

[B14] Ben Salah A Fendri K Gargouri Y La lipase de *Rhizopus oryzae*: production. purification et caractéristiques biochimiques Revue Française Corps Gras 1994 4 5 133 137

[B15] Ghamgui H Karra-Châabouni M Gargouri Y 1-Butyl oleate synthesis by immobilised lipase from *Rhizopus oryzae*: a comparative study between n-hexane and solvent-free system Enzyme and Microbial Technology 2004 35 355 363 10.1016/j.enzmictec.2004.06.002

[B16] Box GEP Hunter WG Hunter JS: Statistics for experiments 1978 New York: John Wiley and Sons 291 334

[B17] Khuri AI Cornell JA Response surfaces: design and analyses Oven DB 1987 New York: Marcel Dekker Inc 8 9

[B18] Thiagarajah T Refining of palm and palm kernel oils. Selected readings on palm oil for participants of palm oil familiarization programme 1992 PORIM. Ministry of Primary Industries. Malaysia

[B19] Rathelot J Julien R Canioni P Coereli C Sarda L Studies on the effect of bile salt and colipase on enzymatic lipolysis. Improved method for the determination of pancreatic lipase and colipase Biochimie 1975 57 1117 1122 1222120 10.1016/s0300-9084(76)80572-x

[B20] Keng PS Basri M Zakaria MRS Abd Rahman MB Ariff AB Abd Rahman RNZ Salleh AB Newly synthesized palm esters for cosmetics industry Industrial crops and products 2009 29 37 44 10.1016/j.indcrop.2008.04.002

[B21] Pedhazur EJ Multiple regression in behavioral research: Explanation and prediction 1982 2 New York: Holt, Rinehart and Winston

[B22] Myers RH Response surfaces methodology 1971 Boston. USA: Allyn and Bacan Inc 67 125

[B23] Yahya ARM Anderson WA Moo-Young M Ester synthesis in lipase-catalyzed reactions Enzyme and Microbial Technology 2001 29 122 128 10.1016/S0141-0229(01)00356-8

[B24] Watanabe Y Pinsirodom P Nagao T Yamauchi A Kobayashi T Nishida Y Takagi Y Shimada Y Conversion of acid oil by-produced in vegetable oil refining to biodiesel fuel by immobilized Candida antarctica lipase Journal of Molecular Catalysis B: Enzymatic 2007 44 99 105 10.1016/j.molcatb.2006.09.007

[B25] Chowdary GV Ramesh MN Prapulla SG Enzymic synthesis of isoamyl isovalerate using immobilized lipase from Rhizomucor miehei: amultivariate analysis Process Biochemistry 2000 36 331 339 10.1016/S0032-9592(00)00218-1

[B26] Laane C Boeren S Vos K Veeger C Rules for optimization of biocatalysis in organic solvents Biotechnology and Bioengineering 1987 30 81 87 10.1002/bit.260300112 18576586

[B27] Yan Z Xiao-Mei W Christopher BW Jing Q Li-Min Z Dual response surface-optimized process for feruloylated diacylglycerols by selective lipase-catalyzed transesterification in solvent free system Bioresource Technology 2009 100 12 2896 2901 10.1016/j.biortech.2009.01.042 19254838

[B28] Karboune S Safari M Lue BM Yeboah FK Kermasha S Lipase-catalyzed biosynthesis of cinnamoylated lipids in a selected organic solvent medium Journal of Biotechnology 2005 119 281 290 10.1016/j.jbiotec.2005.03.012 15899531

[B29] Keng PS Basri M Abd Rahman MB Salleh AB Abd Rahman RNZ Ariff AB Optimization of palm-based wax esters production using statistical experimental designs Journal of Oleo Science 2005 54 10 519 528 10.5650/jos.54.519

